# Risk Factors and Prognostic Significance of Retropancreatic Lymph Nodes in Gastric Adenocarcinoma

**DOI:** 10.1155/2015/367679

**Published:** 2015-01-08

**Authors:** Lian Xue, Xiao-Long Chen, Wei-Han Zhang, Kun Yang, Xin-Zu Chen, Bo Zhang, Zhi-Xin Chen, Jia-Ping Chen, Zong-Guang Zhou, Jian-Kun Hu

**Affiliations:** Department of Gastrointestinal Surgery, West China Hospital, Sichuan University, No. 37 Guo Xue Xiang Street, Chengdu, Sichuan 610041, China

## Abstract

*Background*. The studies on risk factors and metastatic rate of retropancreatic (number 13) lymph nodes in gastric adenocarcinoma were few and the results were still controversial. The aim of this study was to elucidate risk factors and prognostic significance of number 13 lymph nodes in gastric adenocarcinoma. *Method*. From January 2000 to December 2011, 114 patients who underwent gastrectomy with number 13 lymph nodes dissection were enrolled and followed up to January 2014. Patients were grouped according to whether number 13 lymph nodes were positive or negative. *Results*. The metastatic rate of number 13 lymph nodes was 22.8%. In multivariate analysis, pT stage (*P* = 0.027), pN stage (*P* = 0.005), and number 11p (*P* = 0.015) lymph nodes were independent risk factors of positive number 13 lymph nodes. In all patients (*P* < 0.001) and subpopulation with TNM III stage (*P* = 0.007), positive number 13 lymph nodes had significantly worse prognosis than those of patients with negative number 13 LNs in Kaplan-Meier analysis. *Conclusion*. Number 13 lymph nodes had relatively high metastatic rate and led to poor prognosis. pT stage, pN stage, and number 11p lymph nodes were independent risk factors of positive number 13 lymph nodes.

## 1. Introduction

Gastric cancer is one of the most common causes of cancer death in the world, especially in Asian countries [1–3]. Surgery combined with adjuvant chemoradiotherapy is considered to be the main treatment for gastric adenocarcinoma (GAC). Although surgery has been ameliorated in order to cure GAC, patients with adenocarcinoma still have high recurrence rate and poor prognosis. GAC is considered as a disease with a relatively high propensity of lymph nodes (LNs) metastasis, and LNs metastasis is confirmed to be one of the independent prognostic factors of patients who underwent gastrectomy [4–6]. Thus, standard dissection of LNs is considered vital to achieve curative effect [7]. Lymphadenectomy is an important procedure during gastrectomy. However, the optimal extent of lymphadenectomy has still been under debate for a long time. Several clinical trials had been carried out to compare advantages and disadvantages between D1 and D2 lymphadenectomy for primary adenocarcinoma. Dutch trial demonstrated that, in long-time outcomes (15 years), patients with D2 lymphadenectomy had higher overall survival rate and lower recurrence rate than patients with D1 lymphadenectomy [8]. At present, D2 lymphadenectomy has been widely accepted as the standard therapy method for advanced gastric cancer [9–11].

Number 13 LNs are located at the posterior aspect of head of pancreas. According to the Japanese gastric cancer treatment guidelines [12], number 13 LNs were not required to be dissected during standard D2 lymphadenectomy. The Japanese guideline also clarified metastasizing to number 13 LNs as the distant metastasis. Previous studies had discovered that lower-third advanced gastric cancer was prone to having positive number 13 LNs [13]. Metastatic rate of number 13 LNs was reported from 2.53% to 20.8% in primary GAC [14–17]. Some studies reported that metastasis of number 13 LNs was correlated with poor prognosis [14–17]. However, these studies were still few and need further researches.

In this study, we focused on the role of number 13 LNs in primary GAC and sought to compare the differences of clinicopathological characteristics and prognosis between patients with positive or negative number 13 LNs in GAC.

## 2. Methods

### 2.1. Patients

We retrospectively collected patients who were diagnosed with GAC and underwent gastrectomy plus number 13 LNs dissection from January 2000 to December 2011 in the Department of Gastrointestinal Surgery, West China Hospital, Sichuan University. There was no limitation of gender and age. Patients who had previously undergone neoadjuvant chemoradiotherapy were excluded. We divided those patients into two groups according to the metastatic status of number 13 LNs: number 13 LNs (+) and number 13 LNs (−).

### 2.2. Surgical Treatment

Gastrectomy plus lymphadenectomy was the mainstay treatment for GAC. D2/D2+ lymphadenectomy was routinely performed, while D1+ lymphadenectomy was selectively used in early gastric cancer. Number 13 LNs were dissected when these LNs were enlarged or head of pancreas was invaded. Dissection of number 13 LNs was performed as the following procedures: (1) cut lateral peritoneum attached to duodenum and pay attention to preserve gastroduodenal artery; (2) separate the loose connective tissue behind duodenum and head of pancreas to expose the posterior aspect of the pancreas; (3) find number 13 LNs and dissect the LNs with connective tissue completely.

### 2.3. Clinicopathological Features

Clinicopathological characteristics such as tumor size and tumor location, differentiation grade, and pathological TNM stage were recorded according to the Japanese classification of gastric carcinoma by JGCA [18].

Surgical related parameters including operation methods, operation time, intraoperative blood loss, and postoperative complications were recorded. Regarding the close relationship of number 13 LNs and M stage, we defined M0 as no other distant metastases besides positive number 13 LNs and M1 as other distant metastases besides positive number 13 LNs in this study. In addition, pTNM stage was also revised according to M stage we defined.

### 2.4. Follow-Up

Regular outpatient visit was the first choice and follow-up information was updated until January 2014. Telephones and mails were adopted as two main supplementary follow-up methods. During the first 2 years after surgery, follow-up was carried out every 3–6 months, every 6–12 months for next 3–5 years, and then annually [19]. The main reasons for the loss of follow-up were the change of phone number or home address and refusal of reexamination in our hospital.

### 2.5. Statistical Analysis

Continuous variable was presented as mean ± standard deviation and analyzed by Student's *t*-test and Rank Sum test. Rank Sum test and Chi-square test were used to conduct statistical analysis for categorical variable. Life table was used to calculate survival rates. Log-rank test and Cox regression test were used to analyze univariate and multivariate prognostic factors and significance, respectively. Two-tailed *P* value less than 0.05 was considered as statistical significance. All the statistical analyses were performed by statistical software SPSS 16.0 (SPSS, Chicago, IL, USA).

## 3. Results

### 3.1. Demography of Patients

In this study, 114 patients were included with 26 (22.8%) patients in number 13 LNs (+) group and 88 (77.2%) patients in number 13 LNs (−) group. Laparotomy was carried out for 110 patients, and laparoscopy assisted surgery for four patients in whom two patients received conversion to laparotomy. Surgeons decided whether to perform other organ combined dissections according to the situation of cancer invasion and other independent diseases like cholesterol gallstone. Nine (9/114, 7.9%) patients underwent gastrectomy combined with other organs dissections in our study (Table 1).

### 3.2. Metastasis of Number 13 LNs

The average number of dissected LNs was 26.2 ± 12.0 (3–59), and metastatic LNs were 7.6 ± 9.6 (0–49) in all patients. Metastatic rate of number 13 LNs was 22.8% in our study. The highest metastatic rates of regional LNs were number 3 LNs (53.5%), number 6 LNs (49.4%), and number 7 LNs (34.0%) in our study.

### 3.3. Clinicopathological Characteristics Relationship

The clinicopathological characteristics and pathological stage of patients in number 13 LNs (+) and number 13 LNs (−) group were summarized in Table 1. The differentiation grade (*P* = 0.011), macroscopic type (*P* = 0.026), tumor size (*P* = 0.015), pT stage (*P* < 0.001), pN stage (*P* < 0.001), M stage (*P* < 0.001), and pTNM stage (*P* < 0.001) were significantly correlated with metastasis of number 13 LNs. The results indicated that poor differentiation grade, macroscopic types III-IV, tumor size (>5 cm), pT3-T4, pN3, and M1 were associated with positive number 13 LNs. Number 13 LNs (+) group seemed to have more advanced clinicopathological stage when compared with number 13 LNs (−) group. There was no significant difference between these two groups in other clinicopathological factors such as longitudinal location (*P* = 0.560).

The adjacent structures invasion was found in 12 (12/114, 10.5%) patients, with two spleen invasions, one liver invasion, eight pancreas invasions, and one colon invasion. The rate of adjacent structures invasion in number 13 LNs (+) group differed from number 13 LNs (−) group. The results showed that number 13 LNs (+) group had more pancreas invasions than number 13 LNs (−) group (*P* < 0.001). With respect to duodenal invasion, there was no significant difference between number 13 LNs (+) group and number 13 LNs (−) group (*P* = 0.194).

Metastases of number 1 LNs (*P* = 0.033), number 3 LNs (*P* = 0.002), number 7 LNs (*P* = 0.003), number 8a LNs (*P* = 0.001), number 11p LNs (*P* < 0.001), and number 12a LNs (*P* = 0.010) were significantly correlated with metastasis of number 13 LNs in Chi-square test. In logistic regression analysis (Table 2), metastasis of number 13 LNs was significantly associated with pT stage (*P* = 0.027), pN stage (*P* = 0.005), and number 11p (*P* = 0.015).

### 3.4. Operation, Complications, and Mortality

Operation variables were shown in Table 3. There were no significant differences in average operation time (*P* = 0.639) and perioperative blood transfusion rate (*P* = 0.583) between two groups. The total number of harvested LNs was 23.4 ± 13.3 in number 13 LNs (+) group and 27.1 ± 11.6 in number 13 LNs (−) group (*P* = 0.170), and positive LNs were 15.0 ± 13.0 and 5.5 ± 7.2 in number 13 LNs (+) group and number 13 LNs (−) group (*P* < 0.001), respectively. Blood loss was more in number 13 LNs (+) group than in number 13 LNs (−) group (208.1 ± 99.8 mL versus 159.1 ± 70.0 mL, *P* = 0.032).

No patients died within one month after operation. Postoperative complications occurred in eighteen patients, with five (5/26, 19.2%) patients in number 13 LNs (+) group and thirteen (13/88, 14.8%) in number 13 LNs (−) group (*P* = 0.584). The postoperative hospital days were longer in number 13 LNs (+) group than those in number 13 LNs (−) group (15.4 ± 13.2 days versus 10.6 ± 5.6 days, *P* = 0.081).

### 3.5. Survival

Ninety-six patients (96/114, 84.2%) were followed up and analyzed in prognosis with median survival time of 64.7 (2.4–138.9) months. None of patients died accidently till follow-up end time. For all 96 patients enrolled in the survival analysis, the 1-, 2-, and 3-cumulative overall survival rates were 75%, 66%, and 56%, respectively. Three-year overall survival rates were 78.3% and 59.1% in number 13 LNs (−) and number 13 LNs (+) groups, respectively.

**Figure 1:**
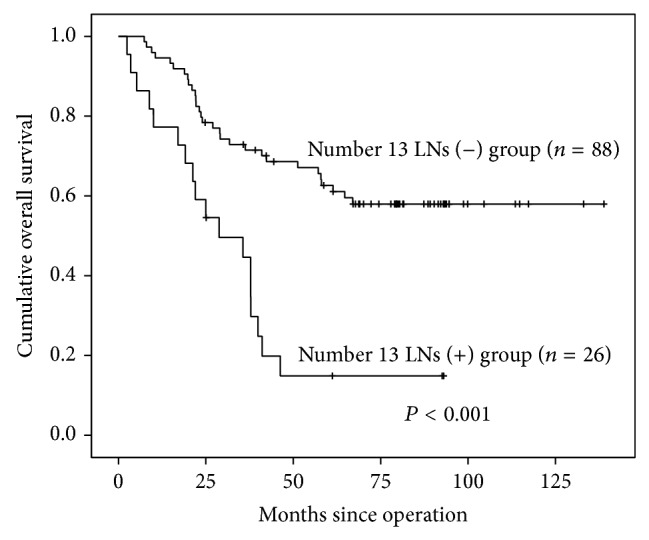
Survival curves of number 13 LNs (+) and number 13 LNs (−) groups by Kaplan-Meier analysis.

Univariate and multivariate analyses for prognostic factors were shown in Table 4. Higher survival rate was shown in number 13 LNs (−) group when compared with number 13 LNs (+) group by Kaplan-Meier analysis (Figure 1, *P* < 0.001). In Kaplan-Meier analysis, tumor size, macroscopic type, pT stage, pN stage, M stage, pTNM stage, and number 13 LNs status were also significantly associated with prognosis (Table 4). Regarding regional LNs, number 3 LNs (*P* = 0.002), number 4d LNs (*P* = 0.001), number 6 LNs (*P* < 0.001), number 7 LNs (*P* = 0.042), number 8a LNs (*P* < 0.001), number 9 LNs (*P* = 0.023), and number 10 LNs (*P* < 0.001) were obviously correlated with prognosis, while number 1 LNs (*P* = 0.999), number 2 LNs (*P* = 0.192), number 5 LNs (*P* = 0.774), number 11p LNs (*P* = 0.068), number 11d LNs (*P* = 0.661), and number 12a LNs (*P* = 0.331) were not correlated with prognosis. Moreover, clinicopathological characteristics and number 13 LNs were also analyzed in Cox regression (Table 4), which demonstrated that pN stage (*P* = 0.003), M stage (*P* = 0.023), and tumor size (*P* = 0.002) were independently correlated with prognosis.

**Figure 2:**
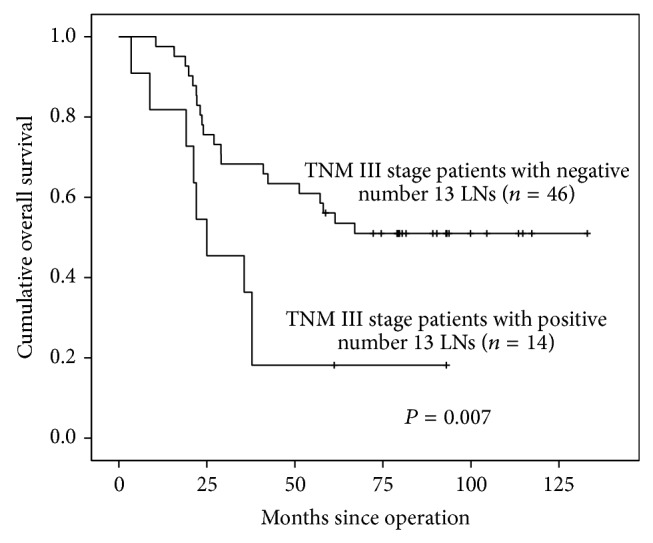
Survival curves of TNM III stage patients with number 13 LNs positive or negative by Kaplan-Meier analysis.

However, patients with positive number 13 LNs seemed to have more advanced TNM stage, larger tumor size, and worse differentiation grade which might relate to worse prognosis. Hence, we focused on patients with TNM III stage. We divided patients with TNM III stage into 2 subgroups according to whether number 13 LNs were positive or negative. Distributions were similar, with regard to TNM stage, differentiation grade, macroscopic type, and tumor size in these two subgroups. In these two subgroups, patients with positive number 13 LNs had significantly worse prognosis (Figure 2, *P* = 0.007).

## 4. Discussion

The lymph nodes metastasis is an important cause for poor prognosis in patients with gastric cancer. In this retrospective study, we analyzed correlated factors with metastasis of number 13 LNs and compared the survival outcomes of patients between number 13 LNs (+) and number 13 LNs (−) groups. The metastatic rate of number 13 LNs was 22.8% in our study, which was higher than 2.53%–20.8% reported in previous researches [14–17]. The possible reason why metastatic rate of number 13 LNs was higher than previous studies might be more advanced tumor stage of patients enrolled in our study. In some researches which reported that metastatic rates of number 13 LNs were 2.53%–9%, respectively, there were no TNM IV stage patients or only 14.2% TNM IV stage patients [14–16]. But in our study, the ratio of TNM IV stage patients was 29.8%, and these patients seemed to have more positive number 13 LNs; therefore, the metastatic rate of number 13 LNs was much higher in our study than previous researches.

We also analyzed metastatic rate of number 13 LNs with clinicopathological characteristics and found that tumor size, differentiation grade, and macroscopic type were significantly correlated with metastasis of number 13 LNs. The tumor size larger than 5 cm might indicate high possibility of number 13 LNs positive (*P* = 0.015), which resembled the previous study [17]. Patients with positive number 13 LNs seemed to have worse differentiation grade and macroscopic type III/IV. Regarding pTNM stage, it seemed that positive number 13 LNs appeared in patients with more advanced stage, which was similar to the previous report [17]. Different incidence of LNs metastasis was observed in number 1 LNs, number 3 LNs, number 7 LNs, number 8a LNs, number 10 LNs, number 11p LNs, and number 12a LNs between number 13 LNs (+) group and number 13 LNs (−) group. The intricate interactions among LNs around stomach might explain these results to some extent. Number 13 LNs had close relationship with other lymph nodes such as number 7 LNs and number 8a LNs. It was reported that number 13 LNs had closed interaction with number 12 LNs and number 14 LNs [20]. It was possibly due to the communicating branches of lymphatic vessels among regional LNs. In addition, logistic regression in our research confirmed that pT stage (*P* = 0.027), pN stage (*P* = 0.005), and number 11p (*P* = 0.015) LNs were independent risk factors of positive number 13 LNs.

Number 13 LNs are located behind head of pancreas and adjacent to duodenum. Therefore, patients suffered more pancreas invasion (26.9%) in number 13 LNs (+) group. Some study found that the incidence of number 13 LNs metastasis was higher in the advanced gastric cancer with duodenum invasion group (23.9% versus 7.0%, *P* < 0.0001), which elucidated close relationship between duodenum and number 13 LNs [21]. However, in our study, duodenum invasion was not significantly correlated with metastasis of number 13 LNs (*P* = 0.194). The possible reason might be that the number of patients with duodenum invasion in our study was small (*n* = 7).

During operation, patients in number 13 LNs (+) group seemed to lose more blood during operation than number 13 LNs (−) group (*P* = 0.032). This might be because more patients with pN2-N3 stage were in number 13 LNs (+) group, which meant more metastasis LNs in this group. Metastasis LNs might compress or even invade vessels which led to vessels bleeding upon dissection. And it is also more difficult to dissect all LNs of patients with pN2-N3 stage. Therefore, during operation, it might cause more blood loss.

Previous research had revealed prognosis significance of number 13 LNs, reporting that patients with positive number 13 LNs had poor prognosis [17]. Our study supported this result through Kaplan-Meier curve which showed that patients with positive number 13 LNs had worse prognosis than negative group (*P* < 0.001), although number 13 LNs was not the independent prognostic factors in Cox regression. The result from Cox regression indicated that pN stage (*P* = 0.003), M stage (*P* = 0.023), and tumor size (*P* = 0.002) were independently correlated with prognosis. However, the worse prognosis in number 13 (+) subgroup may be due to the reason that patients in this group had more advanced tumors. Hence, we divided patients with TNM III stage into number 13 (+) and number 13 (−) subgroups which were more homogenous, and we found that patients with positive number 13 LNs still had worse prognosis. Our result demonstrated that positive number 13 LNs contributed to worse prognosis.

Some retrospective studies had discussed whether to dissect number 13 LNs during gastrectomy [14–17, 20, 21]. Some study held the opinion that D2 plus number 13 LNs dissection for clinical stages III/IV gastric cancer had favorable survival outcomes and concluded that dissection of number 13 LNs in D2 gastrectomy was safe [14, 15]. However, some surgeons performed D3 lymphadenectomy which dissect all third-tier lymph nodes and found that morbidity and mortality rates were higher than those for D2 lymphadenectomy [22, 23]. Japanese guideline excluded number 13 LNs dissection during standard D2 lymphadenectomy, and, in our study, we only compared survival outcomes between patients with or without positive number 13 LNs. The sample of patients enrolled in our study was also small. Although our study found that positive number 13 LNs led to poor prognosis, whether to dissect number 13 LNs during gastrectomy still needed further exploration.

## 5. Conclusion

In conclusion, positive number 13 LNs were usually correlated with more advanced pathological stage and larger tumor size. Positive number 13 LNs might predict poor prognosis although more researches with larger sample size are fervently expected.

## Figures and Tables

**Table 1:** Comparison of clinicopathological characteristics and pathological stage of number 13 LNs (+) and number 13 LNs (−) groups.

	Characteristics	Number 13 LNs (+)	Number 13 LNs (−)	*P* value
		*n* = 26 (%)	*n* = 88 (%)	
Age	Mean ± SD	56.7 ± 9.9	55.6 ± 12.6	0.685
	≥60 yrs	12 (46.2)	35 (39.8)	
	<60 yrs	14 (53.8)	53 (60.2)	

Gender	Male	21 (80.8)	58 (65.9)	0.149
	Female	5 (19.2)	30 (34.1)	

Longitudinal location	U	5 (19.2)	10 (11.4)	0.560
	M	4 (15.4)	17 (19.3)	
	L	17 (65.4)	61 (69.3)	

Differentiation grade	Well/moderate	0 (0)	17 (19.3)	0.011
	Poor/undifferentiated	26 (100)	71 (80.7)	

Macroscopic type	Types 0–2	8 (30.8)	49 (55.7)	0.026
	Types 3–4	18 (69.2)	39 (44.3)	

Tumor size	Mean ± SD	6.1 ± 2.2	5.1 ± 3.2	0.015
	≤2 cm	1 (3.8)	10 (11.4)	
	2–5 cm	9 (34.6)	49 (55.7)	
	5–8 cm	14 (53.8)	19 (21.5)	
	>8 cm	2 (7.8)	10 (11.4)	

Adjacent structures invasion	No	18 (69.2)	84 (95.6)	<0.001
	Spleen	1 (3.8)	1 (1.1)	
	Liver	0 (0)	1 (1.1)	
	Pancreas	7 (27.0)	1 (1.1)	
	Transverse colon	0 (0)	1 (1.1)	

Duodenum invasion	Positive	3 (11.5)	4 (4.5)	0.194
	Negative	23 (88.5)	84 (95.5)	

T stage	T1	1 (3.8)	14 (15.7)	<0.001
	T2	0 (0)	13 (15.7)	
	T3	17 (65.4)	58 (65.2)	
	T4	8 (30.8)	3 (3.4)	

N stage	N0	0 (0)	26 (29.2)	<0.001
	N1	0 (0)	11 (13.5)	
	N2	8 (30.8)	25 (28.1)	
	N3	18 (69.2)	26 (29.2)	

M stage	M0	15 (57.7)	79 (89.9)	<0.001
	M1	11 (42.3)	9 (10.1)	

TNM stage	I	0 (0)	14 (15.7)	<0.001
	II	1 (3.8)	19 (22.5)	
	III	14 (53.8)	46 (51.7)	
	IV	11 (42.4)	9 (10.1)	

LNs: lymph nodes.

SD: standard deviation.

**Table 2:** Multivariate analysis of number 13 LNs metastasis.

	Parameter estimate	SE	Adjusted OR	95% CI	*P* value
Tumor size					0.957
Differentiation grade					0.094
Macroscopic type					0.322
T stage	1.234	0.557	3.434	1.152–10.235	0.027
N stage	1.110	0.394	3.036	1.403–6.566	0.005
M stage					0.146
Number 1					0.273
Number 3					0.056
Number 7					0.054
Number 8a					0.237
Number 10					0.999
Number 11p	3.080	1.272	21.750	1.798–263.106	0.015
Number 12a					0.347

Adjusted OR estimated by the Cox model.

LNs: lymph nodes.

95% CI: 95% confidence interval.

**Table 3:** Comparison of operation and complications of number 13 LNs (+) and number 13 LNs (−) groups.

	Characteristics	Number 13 LN (+)	Number 13 LNs (−)	*P* value
		*n* = 26 (%)	*n* = 88 (%)	
Operation methods	R0	24	85	0.320
	R1/R2	2	3	

Surgical methods	Distal gastrectomy	12	61	0.079
	Proximal gastrectomy	3	8	
	Total gastrectomy	11	19	

Lymph node situation	Positive LNs	15.0 ± 13.0	5.5 ± 7.2	<0.001
	Harvested LNs	23.4 ± 13.3	27.1 ± 11.6	0.170

Operation time (min)	Mean ± SD	248.5 ± 72.0	241.6 ± 58.9	0.639

Intraoperative blood loss (mL)	Mean ± SD	208.1 ± 99.8	159.1 ± 70.0	0.032

Perioperative blood transfusion	Yes	14 (53.8)	42 (47.7)	0.583
	No	12 (46.2)	46 (52.3)	

Postoperative complications	Yes	5 (19.2)	13 (14.8)	0.584
	No	21 (80.8)	75 (85.2)	

Postoperative hospital stay (days)	Mean ± SD	15.4 ± 13.2	10.6 ± 5.6	0.081

LNs: lymph nodes.

SD: standard deviation.

**Table 4:** Univariate and multivariate Cox analysis for prognostic factors.

Risk factors	Categories	Three-year overall survival rate (%)	Univariate analysis *P* value	Multivariate analysis		
				*P* value	OR	95% CI
Age (years)	≤65	82	0.757			
	>65	50				

Gender	Male	65	0.390			
	Female	75				

Differentiation grade	Well/moderate	71	0.137			
	Poor/undifferentiated	54				

Macroscopic type	Types 0–2	72	0.003	0.094		
	Types 3–4	42				

Tumor size	≤5 cm	73	<0.001	0.002	1.849	1.262–2.710
	>5 cm	33				

T stage	T1-T2	81	0.003	0.370		
	T3-T4	50				

N stage	N0	90	<0.001	0.003	1.808	1.227–2.666
	N+	48				

M stage	M0	65	<0.001	0.023	2.218	1.119–4.396
	M1	14				

TNM stage	I-II	84	<0.001			
	III-IV	45				

Number 13 LNs status	Positive	69	<0.001	0.313		
	Negative	15				

## Abbreviations

GAC: Gastric adenocarcinoma
LNs: Lymph nodes.
